# The 7-step “P” technique: a novel manual push method for high-quality OCT imaging in severe coronary stenosis

**DOI:** 10.3389/fcvm.2025.1621424

**Published:** 2025-07-31

**Authors:** Jincheng Guo, Saiying He, Jia Zhou, Hao Liu, Zixuan Li, Zijing Liu, Senhu Wang, Haotian Wang

**Affiliations:** ^1^ Department of Cardiology, Beijing Luhe Hospital, Capital Medical University, Beijing, China; ^2^ Emergency Department, Beijing Luhe Hospital, Capital Medical University, Beijing, China

**Keywords:** catheterization, contrast injection, optical coherence tomography, stenosis, acute coronary syndrome (ACS), percutaneous coronary intervention (PCI)

## Abstract

**Background:**

Achieving optimal optical coherence tomography (OCT) imaging in patients with severe coronary stenosis is challenging because of the catheter-induced restriction of distal contrast flushing within the lesion.

**Aims:**

To evaluate the effectiveness of manual contrast injection followed by OCT catheter advancement in improving image clarity in patients with severe coronary stenosis.

**Methods:**

This single-centre observational study included 60 patients with acute coronary syndrome who demonstrated antegrade thrombolysis in myocardial infarction (TIMI) flow ≥2 on coronary angiography before the OCT catheter was passed through the severe coronary lesions. Catheter advancement resulted in TIMI 0–1 flow. A 7-step “P” technique was developed and implemented to optimise OCT imaging clarity, using manual contrast injection followed by catheter advancement distal to the region of interest (ROI). Image quality was evaluated using a semi-quantitative scoring system to assess the number of quadrants (0–4) in which the vessel walls were clearly visualised.

**Results:**

A total of 1,722 OCT frames were meticulously analysed, with a mean ROI length of 18.99 ± 8.82 mm. This technique consistently produced high-quality images with an average quality score of 3.88 ± 0.38. The proximal lesion segment generally showed a slightly higher image quality compared to the distal segment, with both regions achieving reasonably high clarity scores (3.90 ± 0.28 vs. 3.85 ± 0.45, *p* < 0.001), though the clinical relevance of this difference is minimal. No complications occurred.

**Conclusions:**

The 7-step “P” technique with manual injection is a safe and effective method for acquiring high-quality OCT images in patients with severe coronary artery stenosis, offering a practical alternative in clinical practice.

## Introduction

Intracoronary imaging using optical coherence tomography (OCT) has gained significant attention in recent years because of its higher resolution and faster speed (1). It is recommended as a Class IIa intravascular imaging modality for acute coronary syndrome (ACS) (2) and Class IA for chronic coronary syndrome (3) to guide percutaneous coronary intervention (PCI), underscoring its importance. Traditional OCT imaging requires positioning the catheter 10 mm distal to the region of interest (ROI) and clearing the blood with contrast injection during pullback to obtain high-quality images (1). However, in cases of severe stenosis, achieving clear OCT imaging remains challenging because the wedged catheter limits distal lesion penetration of the contrast medium and results in poor visualisation (4). To overcome this, small balloons are frequently used for vessel predilatation.

To obtain baseline imaging features of untreated tight lesions, innovative techniques such as contrast media infusion followed by catheter push (C-PUSH) or low molecular weight dextran (LMWD) infusion followed by catheter push (D-PUSH), which involve contrast or dextran injection via a power injector, followed by advancement of the OCT catheter through the tight lesion, have shown promising results (5, 6). However, these reports have rarely detailed the preparatory steps preceding catheter advancement, such as the precise retraction position of the OCT catheter after it has crossed and occluded the lesion or the optimal distance it should be advanced, both of which are critical for ensuring procedural reproducibility and high-quality imaging. This lack of standardization becomes particularly relevant when these techniques are applied using manual injection, which may introduce operator-dependent variability. To address this gap, we developed a standardized protocol involving preparatory steps, manual contrast injection, and controlled OCT catheter advancement, aiming to establish a practical and reproducible approach for OCT imaging in lesions with initially preserved TIMI (Thrombolysis in Myocardial Infarction) flow (grade 2 or 3) that became occluded after lesion crossing.

## Methods

### Patients

From December 2020 to January 2023, 60 consecutive patients with ACS who met the inclusion criteria were enrolled. The inclusion criteria were as follows: (1) antegrade TIMI grade 2–3 flow in the target vessel before OCT; (2) the OCT catheter could pass the tight lesions without difficulty; and (3) a puff of contrast showed TIMI grade 0–1 flow after the OCT catheter had passed through the ROI.The exclusion criteria were as follows: lesion morphologies unsuitable for OCT imaging, including ostial lesions, tortuous or heavily calcified vessels, and chronic total occlusions.All patients provided informed consent prior to cardiac catheterization to participate in the study, which was approved by the institutional review board of our institute.

### OCT acquisition techniques

In patients with TIMI grade 2–3 flow after coronary angiography, OCT was performed before any mechanical lesion predilatation or thrombus aspiration. However, in patients with TIMI grade 0–1 antegrade flow, pre-OCT thrombus aspiration or predilation was allowed to restore TIMI grade 2–3 flow. An intravascular Dragonfly^TM^ OPTIS^TM^ imaging catheter or a Dragonfly Duo imaging catheter (Abbott Vascular, Westford, MA, USA) was used for OCT. OCT images were acquired using a commercially available OPTIS Mobile frequency-domain OCT system (Abbott Vascular). After confirmation of the guidewire passing through the target lesion to the distal segment of the vessel, the OCT imaging catheter was advanced at least 10 mm distal to the ROI without any resistance, and a small volume of contrast medium was injected to ensure guide catheter positioning and antegrade flow. In our study, 6 F guiding catheters were routinely used for all procedures.

**Figure 1:**
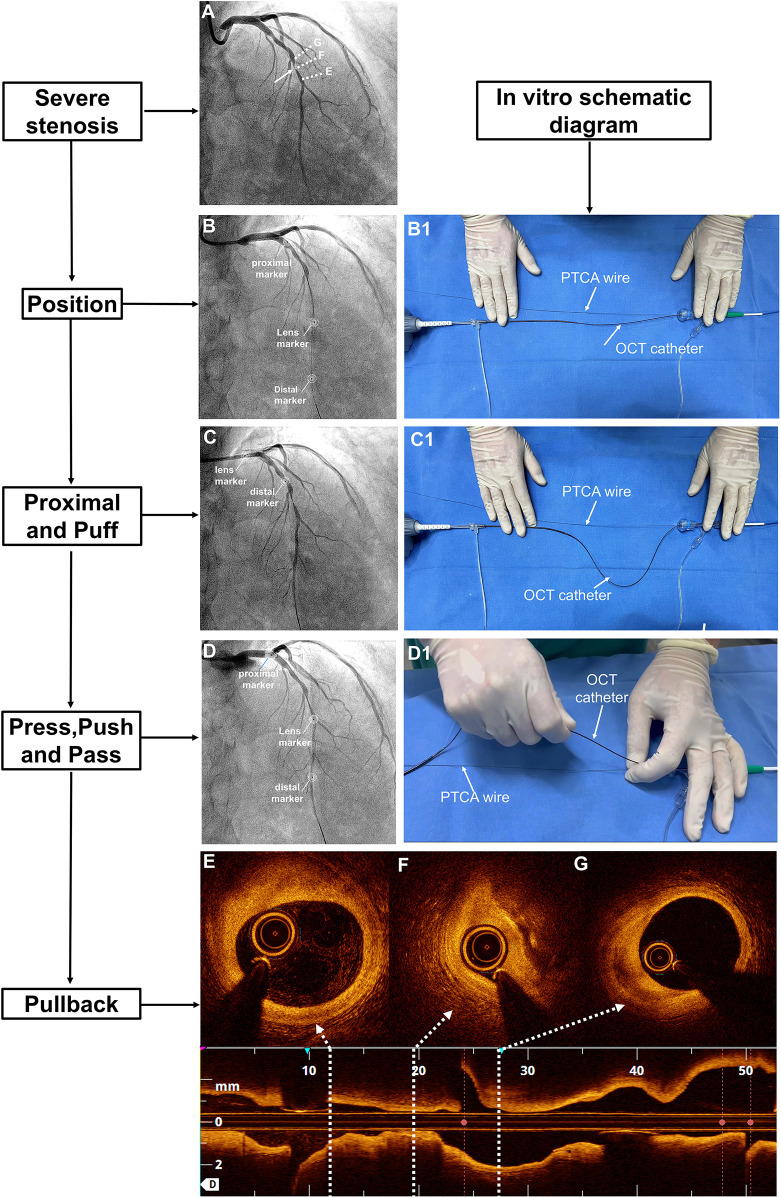
The 7-step “P” technique for managing OCT-induced flow occlusion in cases of tight lesions. **(A)** Coronary angiography revealed a severe lesion in the midsection of the LAD with TIMI grade 3 flow (white arrow). **(B)** After advancing the OCT catheter beyond the ROI, the flow became occluded. Three markers on the OCT catheter were annotated. (B1) The OCT catheter was straightened *in vitro*. **(C)** Upon retracting the OCT catheter to the proximal LAD, TIMI grade 3 flow was restored, while (C1) the distal DOC was stabilised. **(D)** A manual injection of contrast medium was performed before advancing the catheter beyond the ROI, after which (D1) the OCT catheter was straightened again. Finally, **(E–G)** a pullback sequence was initiated. DOC, driver-motor and optical control; LAD, left anterior descending artery; OCT, optical coherence tomography; ROI, region of interest; TIMI, thrombolysis in myocardial infarction.

If there was a TIMI grade 0–1 flow beyond the ROI, the subsequent imaging steps followed the 7 “P” steps: position, proximal, puff, press, push, pass, and pullback. These steps were as follows: The distal OCT lens was **position**ed beyond the ROI, and the OCT catheter was straightened with the driver-motor and optical control (DOC). The OCT catheter was retracted to a point **proximal** to the lesion to ensure that its tip was proximal to the ROI. The full antegrade flow was confirmed by **puff**ing the contrast medium while keeping the DOC in place. The pullback of the FD-OCT system was set to automatic (survey mode: 75 mm at 36 mm/s; 0.2 mm/frame). Then, the operator **press**ed the pedal to activate the angiography machine. Once signalled, the assistant **push**ed the contrast medium beyond the ROI while the operator **pass**ed the OCT catheter through the ROI until it straightened, initiating automatic **pullback** image acquisition (Figure 1). Specifically, manual contrast injection was performed using a standard 12 S control syringe connected to the manifold system. Each manual injection was 12 ml, which corresponds to the maximum capacity of the standard syringe.

This standardized workflow is further demonstrated in a typical case **(**Supplementary Videos S1–S5**)**, which illustrates the complete procedure in a real-world setting. A critical stenosis in the left circumflex (LCX) artery led to complete vessel occlusion upon OCT catheter advancement, after coronary angiography was performed using a 6F guiding catheter **(**Supplementary Videos S1, S2**)**. The subsequent videos document the procedural workflow: Supplementary Video S3 highlights the real-time team coordination during manual administration of a 12 ml contrast injection; Supplementary Video S4 presents the final pullback images; Supplementary Video S5 shows the corresponding angiogram during OCT acquisition.

All procedures were performed by two senior interventional cardiologists, each with over five years of experience in OCT-guided PCI. They were supported by a skilled interventional assistant and an experienced catheterization laboratory technician.

### OCT image analysis

**Figure 2:**
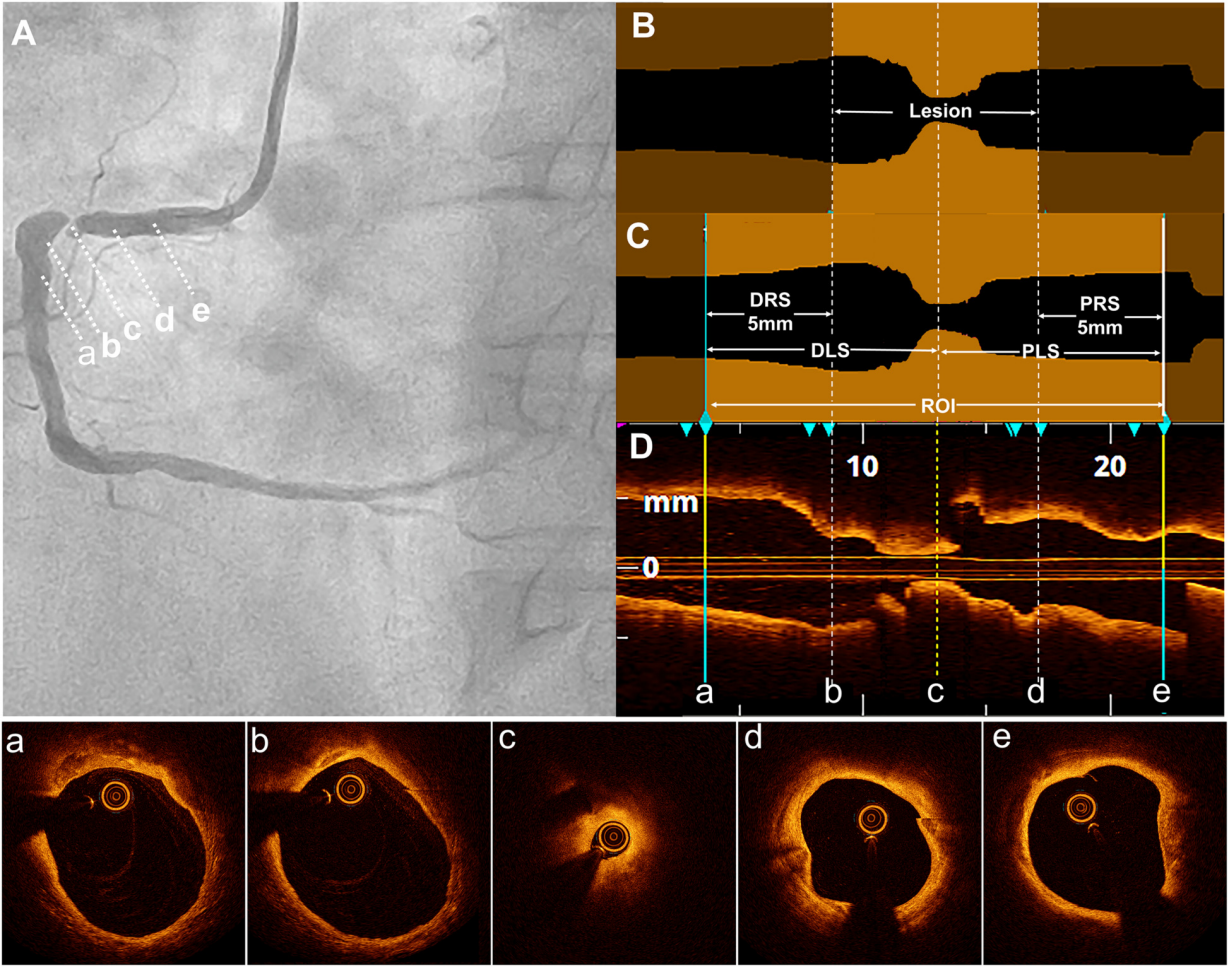
Definition of the lesion, DLS, PRS, and ROI. **(A)** Coronary angiography revealed severe stenosis in the proximal right coronary artery in a typical case. The distal reference segment (a-b), distal lesion (b-c), minimum lumen diameter (MLD) site (c), proximal lesion (c-d), and proximal reference segment (d-e) are shown. **(B)** In the longitudinal optical coherence tomography (OCT) lumen profile, the MLD was located at point (c), with distal and proximal reference points at sites (b) and (d), respectively, where the lumen appeared normal adjacent to the lesion. The lesion length was measured as the distance between these reference points (b and d). **(C)** The distal reference segment (a-b) and proximal reference segment (d,e) were defined as 5-mm segments adjacent to the reference points (b and d). The distal lesion segment (DLS) and proximal lesion segment (PLS) extended between points (a and c) and (c and e), respectively, forming the region of interest (ROI), which included both the DLS and PLS. **(D)** The longitudinal OCT view (L-mode) was accompanied by corresponding cross-sectional images at points a, b, c, d, and e.

The proximal and distal reference sites were defined as sites adjacent to the lesion, proximally and distally, with largest lumen with a percentage plaque area of <50% (7, 8). The proximal reference segment (PRS) and distal reference segment (DRS) were defined as 5-mm segments adjacent to the reference sites and located outside the lesion (9). The lesion length was calculated as the distance between the PRS and DRS. The ROI was defined as the lesion length plus the lengths of the PRS and DRS (10). The proximal lesion segment (PLS) represented the distance from the narrowest point of the lesion or the minimum lumen diameter (MLD) to the proximal end of the PRS. However, the distal lesion segment (DLS) represented the distance from the MLD to the distal end of the DRS (Figure 2).

**Figure 3:**
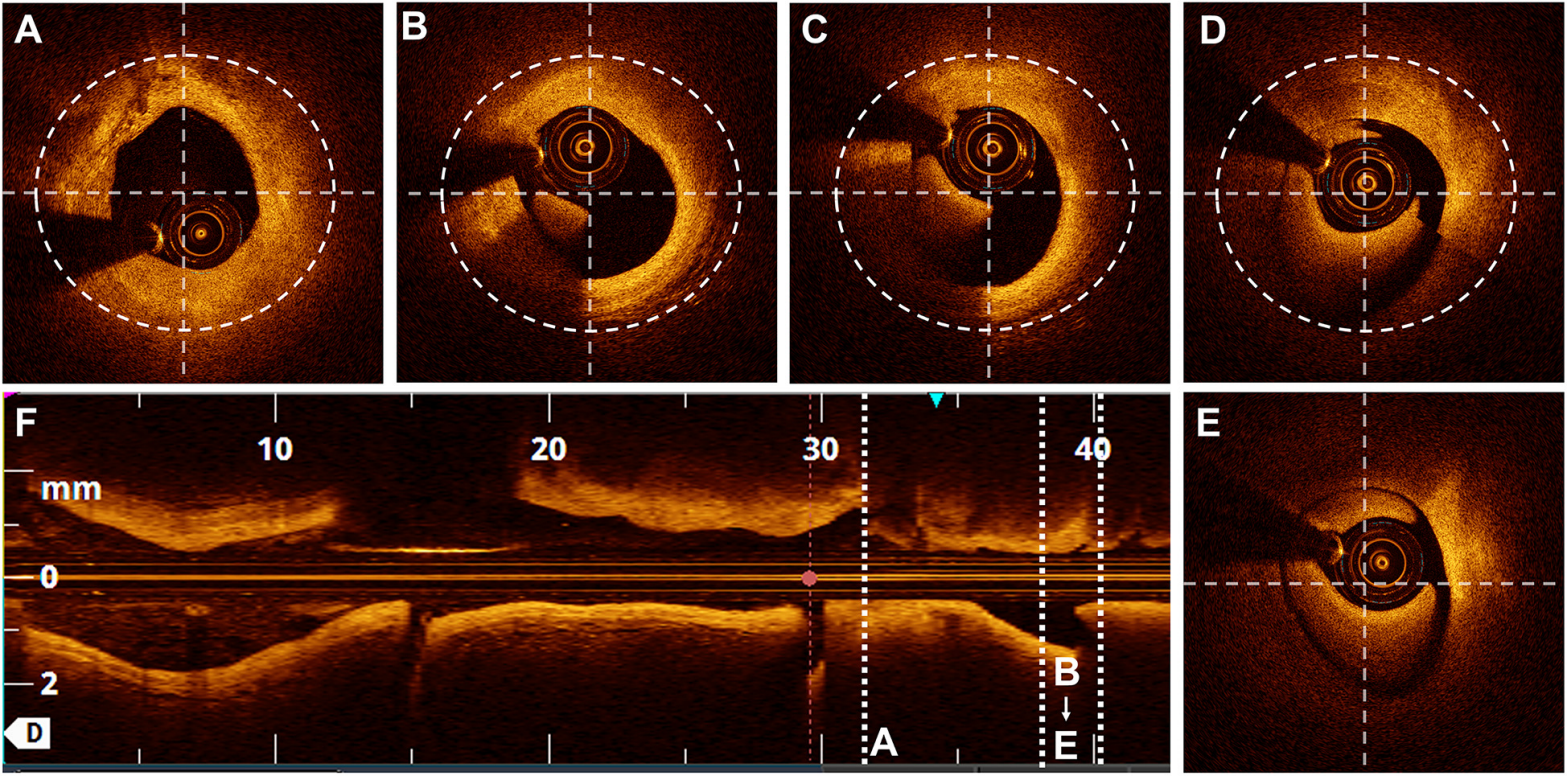
Scores based on the semi-quantitative assessment. Lumen visibility scoring: **(A)** Entire lumen visible-4 points. **(B)** Three-quarters of the circumference visible - 3 points. **(C)** Half of the circumference visible - 2 points. **(D)** One-quarter of the circumference visible - 1 point. **(E)** Less than one-quarter of the circumference visible - 0 points.

A semi-quantitative scale, previously described in the literature (11), was used to evaluate the quality of the OCT cross-sectional image frames. The scoring system was used to evaluate the ability to visualise the lumen as follows: 4 points for the entire circumference, 3 points for three-quarters of the circumference, 2 points for half, 1 point for one-quarter, and 0 points for less than one-quarter of the lumen circumference (Figure 3). Parameters, including the MLD and cross-sectional area, were measured every 1 P along the cross-section.

### Data quality and inter-rater reliability

Data quality was evaluated through independent scoring by two reviewers. The inter-rater reliability was calculated using the kappa coefficient (*κ*), with *κ* = 0.90 indicating excellent agreement between the reviewers, thereby ensuring the reliability and consistency of the scoring process. This high level of agreement ensured the reliability and consistency of the scoring process.

### Statistical analysis

Statistical analyses were performed using SPSS (version 22.0; Chicago, Illinois, USA). Continuous variables are reported as mean ± standard deviation for normally distributed data and as median with interquartile range for non-normally distributed data. Categorical variables are presented as absolute numbers and percentages. Categorical variables were compared using the chi-square test or Fisher's exact test, as appropriate. The level of statistical significance was set at *p* < 0.05.

## Results

The study included 60 lesions from 60 patients, with a mean age of 59.8 ± 14.0 years. Of these patients, 71.7% had ST-segment elevation myocardial infarction. The pre-OCT treatment approaches included thrombus aspiration (45.0%), predilatation with a small balloon (10.0%), and combined approaches (8.3%). The baseline and angiographic characteristics are summarised in Tables 1, 2, respectively.

**Table 1:** Baseline characteristics of the patients.

Variables	Study population (*n* = 60)
Age (years)	59.8 ± 14.0
Males, *n* (%)	44 (73.3)
Body mass index (kg/m^2^)	26.05 ± 2.78
Current or previous smoking, *n* (%)	31 (51.7)
Hypertension, *n* (%)	33 (53.3)
Hyperlipidemia, *n* (%)	16 (26.7)
Diabetes mellitus n, (%)	17 (28.3)
Renal insufficiency, *n* (%)	3 (5.0)
History of stroke, *n* (%)	2 (3.3)
Previous history of MI, *n* (%)	6 (10.0)
Previous history of PCI, *n* (%)	8 (13.3)
Creatine (ml)	74.2 ± 19.3
Clinical presentation, *n* (%)	
UAP	6 (10.0)
NSTEMI	11 (18.3)
STEMI	43 (71.7)
Ejection fraction (%)	62.6 ± 10.8

Values are Mean ± SD, *n* (%), or median (25th, 75th percentiles). PCI, percutaneous coronary intervention; MI, myocardial infarction; UAP,unstable angina; NSTEMI, non-st-elevation myocardial infarction; STEMI, ST-elevation myocardial infarction.

**Table 2:** Procedural characteristics.

Variables	Study population (*n* = 66)
Right radial artery access, *n* (%)	66 (100)
Sheath size 6Fr, *n* (%)	66 (100)
Target vessel, *n* (%)	
LAD	29 (48.3)
LCX	11 (18.3)
RCA	20 (33.3)
Coronary segment, *n* (%)	
Proximal	19 (31.7)
Mid	33 (55.0)
Distal	8 (13.3)
Pre-OCT treatment, *n* (%)	
Thrombus aspiration alone	27 (45.0)
Predilatation alone	6 (10.0)
Thrombus aspiration + predilatation	5 (0.3)
Initial TIMI flow grade, *n* (%)	
0/1	20 (33.3)
2	3 (5.0)
3	37 (61.7)
Pre-OCT flow grade, *n* (%)	
3	60 (100.0)

Values are Mean ± SD, *n* (%). LAD, left anterior descending artery; LCX, left circumflex artery; OCT, optical coherence tomography; RCA, right coronary artery; TIMI, thrombolysis in myocardial infarction.

**Figure 4:**
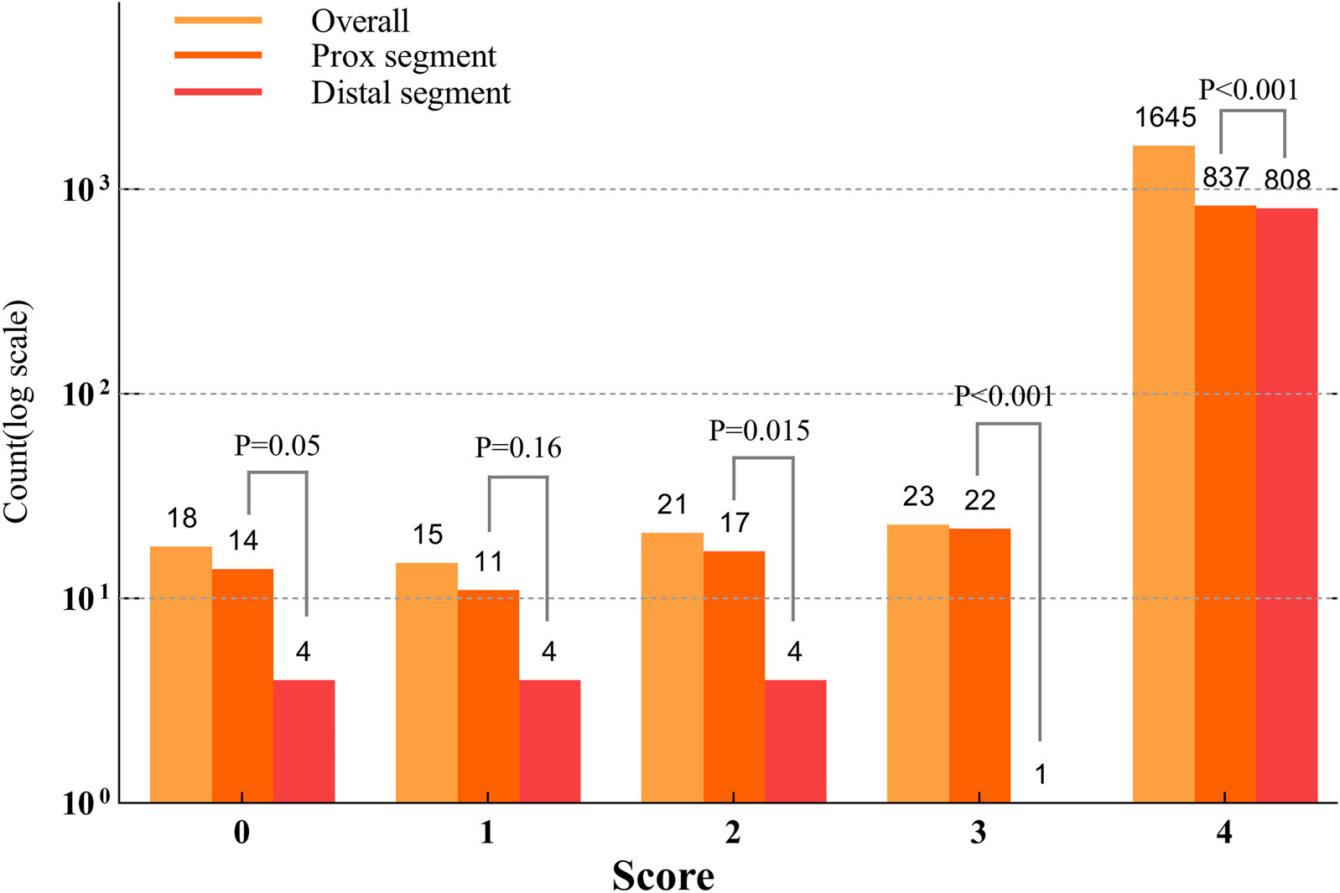
Counts based on the scores across the lesion segments (Log10 scale). The distribution of lesion segment counts across scores from 0 to 4 indicated that the proximal lesion segments had significantly higher scores in 0, 1, 3, and 4 compared to the distal lesion segments (*p* < 0.001). No significant difference was observed for score 2 (*p* = 0.1).

A total of 1,722 OCT frames were analysed, with an average of 28.7 ± 8.84 frames per lesion. Table 3 presents measurements of the reference diameter, MLD, cross-sectional area, ROI length (28.99 ± 8.82 mm), and lesion characteristics. The score distribution was as follows: 1.0% of the frames scored 0, 0.9% scored 1, 1.2% scored 2, 1.3% scored 3, and 95.5% scored 4. The mean overall image quality score was 3.88 ± 0.38, indicating high-quality imaging overall. Although the PLS score was slightly higher than the DLS score(3.90 ± 0.28 vs. 3.85 ± 0.45, *p* < 0.001), both demonstrated excellent image clarity. The overall PLS and DLS scores are shown in Figure 4.

**Table 3:** Detailed OCT analysis.

Variables	Study population (*n* = 60)
Diameter (mm)	
Proximal reference	2.82 ± 0.52
MLD	1.00 ± 0.74
Distal reference	2.55 ± 0.56
CSA (mm^2^)	
Proximal reference	6.47 ± 2.42
Minimum lumen	0.81 ± 0.13
Distal reference	5.38 ± 2.42
Length (mm)	
MLD site to the ostium	32.01 ± 14.54
Lesion length	18.99 ± 8.82
MLD site to proximal reference	8.81 ± 6.41
MLD site to distal reference	10.27 ± 7.02
ROI	28.99 ± 8.81
Lesion characteristics, *n* (%)	
Plaque rupture	16 (26.7)
Non-plaque rupture	44 (73.3)
Thrombus	36 (60.0)
White	22 (36.7)
Red	9 (15.0)
Combination	5 (8.3)

CSA, cross-sectional area; MLD, minimum lumen diameter; ROI, region of interest.

Data quality was ensured through independent scoring by two reviewers, demonstrating excellent inter-rater reliability (*κ* = 0.92). Moreover, no procedure-related complications, such as arrhythmia, embolisation, coronary dissection, or spasm, occurred during OCT catheter imaging.

## Discussion

This study evaluated manual contrast injection via a syringe through the guiding catheter, followed by pushing the OCT catheter to obtain clear OCT images in cases where the antegrade flow was TIMI grade 2 or 3 before the OCT catheter crossed the severe coronary lesions, which were subsequently occluded by the catheter, preventing the contrast medium from effectively flushing out blood for clear imaging. This 7-step “P” technique was demonstrated to be feasible and safe, resulting in high-quality imaging without any procedure-related complications.

### Strategies for OCT in tight lesions

Adequate blood removal requires the consideration of factors such as vessel diameter, tortuosity of the vessel, coaxial positioning of the guiding catheter, contrast flush, type of pullback trigger, optimal synchronisation between flushing and OCT catheter pullback, and the operator's skills (4, 10, 12). Despite addressing these factors, performing OCT for tight coronary lesions remains challenging. The poor imaging quality in these cases is often due to the limited luminal space and inadequate blood clearance. Several strategies have been developed to address these challenges, improve the imaging quality, and ensure successful OCT in difficult scenarios. Balloon predilation is commonly used to widen lesions and ensure vessel clearance after OCT catheter passage (4). However, it may alter lesion morphology, obscure original details, and interfere with the accurate assessment of plaque characteristics, mechanisms (e.g., rupture and erosion), and thrombus presence in patients with ACS (13).

An alternative to predilation is the double injection technique, which involves simultaneous distal OCT catheter injection using a purge connector combined with a proximal flush through the guiding catheter, thereby improving image quality (6). Although effective, the use of a narrow purge lumen for this technique may create excessive pressure, leading to live OCT image distortion and an increased risk of core breakage at high rotation speeds (11). The study reported a total score of 3.1 ± 0.6, lower than the 3.50 ± 0.85 observed in our study using the same scoring scale (6). The C-PUSH or D-PUSH technique uses contrast media or dextran infusion, followed by catheter pushing with an automatic injector for over 4 s for the left coronary artery and 3 s for the right coronary artery (5, 6). Power injections are more reliable for achieving a blood-free lumen (10). In settings where automatic injectors are not available, manual contrast injection represents a feasible and practical alternative. Our study confirmed the safety and efficacy of the C-PUSH technique using manual injection and refined its procedural steps to improve consistency. These refinements enhance the technique's applicability in settings without automatic injectors and support its broader adoption in clinical practice.

### Tips for clear OCT imaging

The 7-step “P” technique represents a significant methodological advancement for OCT imaging in tight lesions, primarily due to its novel, multi-stage preparatory protocol (Steps 1–3: Position, Proximal, Puff), which distinguishes it from direct “place-and-push” methods such as C-PUSH and D-PUSH (5). This step is novel for two key reasons. First, it confirms that flow cessation is iatrogenic and reversible. Second, it enables precise calibration of the pullback distance required to traverse the ROI. This is achieved by retracting the imaging catheter while keeping the DOC stationary until blood flow is restored. This pre-calculation, which is not described in prior techniques, facilitates rapid, targeted, and efficient catheter advancement during the procedure. From a practical standpoint, the manual 7-step “P” technique serves as a valuable complement to approaches using automated injectors, thereby expanding its applicability to a wider range of catheterization laboratories. Its “7P” mnemonic further enhances clinical translation by providing a standardized, reproducible, and easily learned framework that supports broad adoption.

Seamless team coordination is essential for advanced OCT imaging in severe coronary stenosis. Yamaguchi et al. (6) emphasized that precise timing between the operator and technician is critical for success. Similarly, Kobayashi et al. (5) highlighted the steep learning curve of the D-PUSH technique, underscoring the need for synchronized execution. Our method reflects these findings, particularly in the final three steps of the 7-step “P” technique—push (manual injection by the assistant), pass (catheter advancement by the interventional cardiologist), and pullback (trigger activation by the technician). Across all studies, successful imaging relies on close collaboration among the interventional cardiologist, assistant, and technician.

### OCT image quality assessment

OCT image quality assessment commonly uses a semi-quantitative scale for quick subjective evaluation (emphasising vessel clarity, contrast, and diagnostic value) or quantitative analysis, which measures visible arcs at 1 mm intervals to provide objective clarity (5, 11). Another method classifies OCT runs as good, clinically usable, or unusable, based on vessel visualisation, blood clearance, and diagnostic value. Runs categorised as good or clinically usable are considered effective for optimisation (14). In our study, we adopted the semi-quantitative scale described previously (11), offering a balanced approach to image quality assessment that provides reliable diagnostic information without requiring complex measurements. This scale facilitated efficient image evaluation, which was crucial for timely clinical decision-making while ensuring that the OCT image quality met diagnostic standards.

### Limitations

This study had several limitations. First, it was a single-centre, non-randomised, observational study with a limited sample size, and only 6F guiding catheters were used. Second, lesions with severe tortuosity or calcification and those in which the OCT catheter failed to cross the lesion were excluded. Third, the technique has a learning curve that requires close coordination between the operator, assistant, and technician. Finally, our single-center findings warrant broader validation. A logical next step would be to conduct a prospective, multicenter registry to further evaluate the safety, feasibility, and reproducibility of the 7-step “P” technique across a range of operators and clinical institutions.

## Conclusions

This study confirms the safety and adaptability of the 7-step “P” technique with manual injection for OCT imaging, providing a practical alternative for achieving high-quality images in clinical settings.

## Data Availability

The original contributions presented in the study are included in the article/Supplementary Material, further inquiries can be directed to the corresponding authors.

## Supplementary material

Supplementary Video 1 Coronary angiography demonstrating significant stenosis in the target lesion.

Supplementary Video 2 OCT catheter advancement resulted in complete flow obstruction during imaging preparation.

Supplementary Video 3 Demonstration of the coordinated workflow among the operator, assistant, and technician during the procedure.

Supplementary Video 4 Final OCT pullback image revealing detailed lumen morphology and lesion characteristics.

Supplementary Video 5 Angiographic view of the OCT acquisition sequence: contrast injection, catheter advancement, and automated pullback.
